# Compound Comminuted Fracture of the Left Femur, with Much Laceration of the Soft Parts and the Knee Joint Freely Opened

**Published:** 1885-03

**Authors:** Charles F. Pickering

**Affiliations:** Surgeon to the Bristol General Hospital


### COMPOUND COMMINUTED FRACTURE OF THE LEFT FEMUR, WITH MUCH LACERATION OF THE SOFT PARTS AND THE KNEE JOINT FREELY OPENED. By Charles F. Pickering, F.R.C.S., Surgeon to the Bristol General Hospital.

W. H., aged 26, a brass moulder, was knocked down by a tram car and run over. On admission to the hospital on June 20th, 1884, immediately after the accident, the patient was found to have sustained a compound fracture of the femur, just above the condyles, with a lacerated wound, about four inches by six inches in extent, on the posterior surface of the thigh, and with apparent loss of a large piece of skin, thus exposing the muscles, which were bruised and lacerated. This wound communicated freely with the knee joint, and allowed of the finger being passed easily into the joint, across to the other side of the thigh, and down into the popliteal space.

The wound was thoroughly washed out with carbolic lotion 1-20; some small pieces of loose bone were removed; three drainage tubes were inserted, and the parts surrounded by a carbolic gauze dressing.

It was decided (after consultation with my colleagues) that an attempt should be made to save the leg, and to see what conservative surgery could accomplish.

For the first two weeks after the accident the patient's pulse averaged from 80-90, and the temperature from 99°-101°, but on two occasions nearly reaching 102°; from this time the temperature was constantly normal. For the first 18 days the dressings required changing daily on account of the free serous oozing, the following two weeks every other day, and afterwards about once a week.

Although some sloughing of the soft parts occurred, the wound during the whole time was entirely devoid of local inflammatory symptoms. No tension was ever present, and the knee joint healed up as if it had never been opened—I should say better, for there was no swelling or pain at any time, symptoms so common in fractures of the femur low down.

The patient was suffering from a good deal of shock when admitted, and continued to look pale and anxious for about 14 days afterwards, and to show that the discharges and the shock of so severe an accident were telling seriously upon the system; but at the end of a fortnight he began to put on flesh and to look happy and well.

The bone did not unite kindly for the first month; I think this was owing to the frequent movement which changing the dressing entailed; but by Aug. 14th there was good union, and the wound was healing rapidly. A Croft's plaster splint was now substituted for the long Liston splint.

On August 22nd the patient left his bed for the first time.

August 29th. The fracture was firmly united, and slight movement in the knee joint was present.

September 12th. The patient left the hospital, with the leg supported by a Croft's splint. The wound was still dressed with carbolic gauze dressings, but was now quite superficial and healing rapidly. The movement in the joint was increasing. The patient was instructed to come to the hospital weekly to be dressed.

November 29th. The carbolic gauze dressings were left off, and ung. pot. chlor. ordered, with which the patient was instructed to dress the wound daily. The sore was now rather larger than a crown piece, and still healing, notwithstanding the extent of the cicatrix surrounding it. There was fair movement in the joint, and the patient could walk well with the aid of two sticks. The leg was shortened by about half an inch, but was firm and strong. The Croft's splint was left off, as the thigh did not now require support.

*Remarks.*—In many compound fractures of the thigh with open wound of the knee joint, that the leg has been saved without the employment of strict antiseptic precautions is undoubted; but I would maintain that the surgeon who would have tried to save the leg in so serious a case as the above without their most rigid employment would have been bold indeed, as the life of the patient must necessarily be seriously endangered.

The object I had in view was not only to save the leg, but to give the patient a useful joint; in which I fortunately succeeded.

[I must thank the House Surgeon (Mr. J. W. Penny) for his valuable help in the dressing of the case, which wanted most constant care and attention.]

---

